# Relationship between the Reduced Expression of Zinc Finger Protein 668 in Bladder Cancer and Its Invasiveness

**DOI:** 10.3390/ijms24108668

**Published:** 2023-05-12

**Authors:** Yumiko Okuno, Mami Hattori-Kato, Hiroki Tanaka, Akiko Tonooka, Takumi Takeuchi

**Affiliations:** 1 Department of Urology, Japan Organization of Occupational Health and Safety, Kanto Rosai Hospital, 1-1 Kizukisumiyoshi-cho, Nakahara-ku, Kawasaki 211-8510, Japan; 2 Department of Pathology, JR Tokyo General Hospital, 2-1-3 Yoyogi, Sibuya-ku, Tokyo 151-8528, Japan; tanakahiroki19860611@yahoo.co.jp; 3 Department of Pathology, Cancer Institute Hospital of Japanese Foundation for Cancer Research, 3-8-31 Ariake, Koto-ku, Tokyo 135-8550, Japan

**Keywords:** bladder cancer, ZNF668, invasion

## Abstract

The zinc finger protein 668 (*ZNF668*) gene encodes a Kruppel C2H2-type zinc-finger protein with 16 C2H2-type zinc fingers. The *ZNF668* gene functions as a tumor suppressor gene in breast cancer. We histologically analyzed ZNF668 protein expression in bladder cancer and examined mutations of the *ZNF668* gene in 68 cases of bladder cancer. In bladder cancer, the ZNF668 protein was expressed in the nuclei of cancer cells. In bladder cancer with submucosal and muscular infiltration, the expression of ZNF668 protein was significantly lower than that without submucosal and muscular infiltration. Eight heterozygous somatic mutations were detected in exon3 in five cases, and five of the mutations resulted in amino acid sequence mutations. Mutations resulting in amino acid sequence alterations also resulted in lower ZNF668 protein expression in bladder cancer cell nuclei, but no significant association with bladder cancer infiltration was detected. Decreased ZNF668 expression in bladder cancer was associated with submucosal and muscle invasion of cancer cells. Somatic mutations resulting in amino acid mutations in *ZNF668* were found in 7.3% of the bladder cancer cases.

## 1. Introduction

The zinc finger protein 668 (*ZNF668*) gene encodes a Kruppel C2H2-type zinc-finger protein with 16 C2H2-type zinc fingers. According to the Alliance of Genome Resources, November 2021 [1], ZNF668 is predicted to have DNA-binding transcription activator activity, namely RNA polymerase II-specific and RNA polymerase II cis-regulatory region sequence-specific DNA binding activity. The ZNF668 protein is also predicted to be involved in the regulation of transcription by RNA polymerase II and to be active in the nucleus. The three-dimensional structure of the ZNF668 protein predicted by AlphaFold [2] is available [3].

It was previously reported that the *ZNF668* gene was mutated in 11.4% of breast cancers [4]. The ZNF668 protein functions as a tumor suppressor gene in breast cancer by inhibiting the degradation of a prominent cancer suppressor, p53, after binding to MDM2, aiding the stability of p53 in repairing DNA damage [5]. ZNF668 is also associated with chromatin relaxation, which is important in repairing DNA damage [6]. Moreover, ZNF668 knockdown inhibits DNA repair by homologous recombination in breast cancer cells [7]. On the genetic side, Alsaif et al., reported that, in two families, biallelic truncating variants in *ZNF668* resulted in microcephaly, growth deficiency, severe global developmental delay, brain malformation, and distinct facial dysmorphism [8].

Therefore, it is necessary to analyze the expression and gene mutation of *ZNF668* in malignant tumors other than breast cancer. Here, we histologically analyzed the production of ZNF668 protein via *ZNF668* gene expression (hereinafter also referred to simply as “ZNF668 protein expression”) in bladder cancer and examined mutations of the *ZNF668* gene in bladder cancer tissue.

## 2. Results

### 2.1. Immunohistochemistry

**Figure 1:**
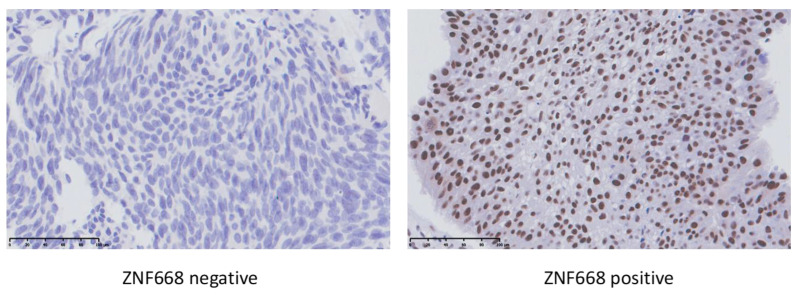
Representative images of ZNF668 immunohistochemistry in bladder cancer. (**Left**): negative staining of the nuclei in bladder cancer cells; (**Right**): positive staining of the nuclei, Scale bars = 100 μm.

In bladder cancer, the ZNF668 protein was expressed in the nuclei of cancer cells (Figure 1), but in a few cases, it was also expressed in the cytoplasm. As shown in Table 1, in bladder cancer with submucosal infiltration, the expression of the ZNF668 protein evaluated by IRS was significantly lower than that without submucosal infiltration. In bladder cancer with muscle invasion, the expression of ZNF668 protein evaluated by IRS was significantly lower than that without muscle invasion.

### 2.2. Structural Analysis of the ZNF668 Gene

Exon1 corresponds to the 5′-untranslated region (5′-UTR). No somatic mutations in exon1 were found in the bladder tumor genome. Regarding the single nucleotide polymorphisms (SNP) in exon1, as shown in Table 2, there was no difference in ZNF668 protein expression in bladder tumors in the G/A heterozygous cases at the 5′-UTR variant rs2303222 site compared with the G/G homozygous cases. There was no association between rs2303222 and bladder cancer invasiveness. In addition, heterozygosity of rs192022056 (G/A) and rs1044370356 (T/C) was found in two cases each.

A part of exon2 corresponds to the 5′-UTR, and the rest encodes a protein-coding region. No somatic mutations in exon2 were found in the bladder tumor genome. As shown in Table 2, there was no difference in ZNF668 protein expression in bladder tumors in the A/G heterozygous cases compared with the A/A homozygous cases at the rs2303223 site, which causes synonymous variation. There was no association between rs2303223 and bladder cancer invasiveness. No other variations were observed in exon2.

**Figure 2:**
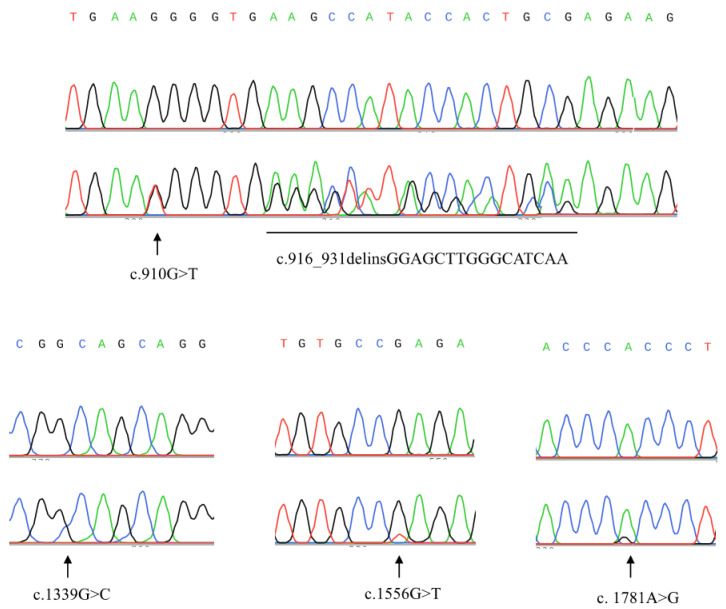
Sanger sequencing data showing somatic mutations in exon3 of *ZNF668* gene. Upper: reference germline sequences; Lower: indicating somatic mutations.

Exon3 consists of a part of a protein-coding region and a 3′-untranslated region. As shown in Table 3 and Figure 2, eight heterozygous somatic mutations were detected in five cases, of which five mutations resulted in amino acid sequence mutations. Mutations resulting in amino acid sequence alterations also resulted in lower ZNF668 protein expression in bladder cancer cell nuclei, but no significant association with bladder cancer infiltration was detected (Table 2). For the SNPs in exon3, synonymous variations rs767895927, rs768496978, and rs2288003 were detected, in one case each, in a heterozygous manner.

## 3. Discussion

In the histological analysis, the decreased ZNF668 protein expression in bladder cancer cell nuclei was associated with submucosal and muscle infiltration of bladder cancer. Therefore, *ZNF668* may act as a tumor suppressor in bladder cancer, as well as in breast cancer. However, the rate of somatic mutations in the exons of the *ZNF668* gene in bladder cancer was not high (7.3%). In this study, a double somatic mutation was detected in one case (Case B), and a triple somatic mutation was detected in another case (Case E). In the former, each mutation resulted in an amino acid mutation, while in the latter case, one mutation resulted in an amino acid mutation, and the other two mutations were present in 3′-UTR. Whether these multiple somatic mutations were in cis or in trans could not be determined by the assay adopted in this study. If *ZNF668* acts as a tumor suppressor, it is assumed that the occurrence of multiple somatic mutations in trans will lead to tumor growth. It is known that genetic mutations in 3′-UTR are associated with protein expression levels [9].

These findings suggest that somatic mutations in exon3 (if there are any) are also associated with decreased ZNF668 protein expression, while the factors responsible for the decreased ZNF668 protein expression in most bladder cancer cases may be mainly due to factors other than somatic mutations in the exon region such as mutations in regulatory regions or epigenetic changes. The tendency for ZNF668 protein expression to be reduced by heterozygous somatic mutations was likely a minor effect, although the possibility that haploinsufficiency or dominant-negative effects partially contributed cannot be ruled out. Activation and silencing of multiple genes are important in the development and progression of cancer. Silencing of cancer-related genes, especially DNA repair genes, often results from hyper-methylation at multiple CpG sites in CpG islands in the promoter regions of genes, in addition to gene mutations [10]. As for the SNPs, relatively frequent variants were detected in exon1 and exon2, but the variants were not associated with reduced ZNF668 protein expression or submucosal or muscle invasiveness of bladder cancer.

The reason why the down-regulation of ZNF668 is associated with cancer invasiveness remains uncertain. According to Zhang et al., decreased ZNF668 expression is associated with cancer infiltration and metastasis in non-small cell lung cancer, and overexpression of ZNF668 results in decreased Snail expression and increased E-cadherin and ZO-1 expression [11]. The decreased expression of Snail is also associated with the action of p53 [12]. Assuming that the ZNF668 is involved in the DNA repair, the decreased expression of the ZNF668 protein in the nuclei of bladder cancer cells may contribute to cancer progression. Further investigation of the relationship between ZNF668 and tumor suppression is needed in future studies. 

In the present study, tumor sampling was performed by trans-urethral resection of the bladder tumors (TUR-Bt), and the presence of carcinoma in situ was not rigorously verified in individual cases. Therefore, the association between ZNF668 and carcinoma in situ cannot be examined in this study.

Similar to a previous report, we observed ZNF668 expression in the cytoplasm of cancer cells in a small number of cases [11], but its significance is unknown at this time. Patients with low expression of ZNF668 protein in the nuclei of bladder cancer cells are at high risk for muscle invasion, which is a poor prognostic factor. Therefore, more intensive follow-up, such as induction of second TUR-Bt, is recommended in such cases. In conclusion, the decreased ZNF668 expression in bladder cancer was associated with submucosal and muscle invasion of cancer cells. Somatic mutations resulting in amino acid mutations in ZNF668 were found in 7.3% of the bladder cancer cases.

## 4. Materials and Methods

In total, 74 patients with bladder cancer (60 males and 14 females) were included in this study. The average age of the patients was 73.0 ± 9.9 years.

### 4.1. Immunohistochemistry

Formalin-embedded tissues from 67 bladder tumors were collected during TUR-Bt and were stained with anti-human rabbit ZNF668 antibody (NBP1-92615, Novus Biologicals) to examine ZNF668 protein expression. Two independent pathologists evaluated the histological staining using the immunore-active score (IRS) [13], and the individual scores were analyzed after averaging.

### 4.2. Structural Analysis of the ZNF668 Gene

Genomic DNA was extracted from bladder tumor tissues that were freshly frozen in liquid nitrogen for 68 cases. Primers were designed for flanking the introns for exons 1, 2, and 3 that compose the *ZNF668* gene. PCR products amplified by pairs of sense and antisense primers were directly sequenced using the same primers. The sense and anti-sense primers for amplification consisted of 5′-gtccttaggtgcaaaagcttccccg-3′/5′-ccg-cagggaaactgaggccagctc-3′ for exon1 (amplicon size 833 bp), 5′-tgaggctttcaggag-tggcgaaggt-3′/5′-ttaccctgagactcaaacccaggcc-3′ for exon2 (818 bp), and 5′-gcagtggggtcac-gttatgggtctg-3′/5′-tgatgcccaaactcccacccattca-3′ for exon3 (1545 bp). If necessary, analysis of the exons of the ZNF668 gene was also performed using germline genomes purified from blood. PCRs were performed in a 50-μL volume containing 0.3 μM sense and antisense primers, 1 m M of MgSO4, 4% DMSO, and 1 unit of KOD-plus-ver.2 polymerase (TOYOBO) as follows: 94 °C for 2 min, 40 cycles (98 °C for 10 s for denaturing, 68 °C for 90 s for annealing and extension) by 2-step PCR.

## 5. Conclusions

The decreased ZNF668 expression in bladder cancer was associated with submucosal and muscle invasion of cancer cells. Somatic mutations resulting in amino acid mutations in ZNF668 were found in 7.3% of the bladder cancer cases.

## Figures and Tables

**Table 1:** Relationship between ZNF668 protein expression and cancer infiltration in bladder cancer.

	Submucosal Invasion		
	Plus	Minus	*p*-Value
IRS	4.34 ± 2.16	6.68 ± 3.23	0.0007
	muscle invasion		
	plus	minus	
IRS	3.46 ± 2.51	6.20 ± 2.92	0.0026

IRS: immunoreactive score, the *p*-values were calculated using the unpaired *t*-test.

**Table 2:** Association of *ZNF668* gene variations with ZNF668 protein expression and invasiveness in bladder cancer.

	rs2303222 (Exon1)			rs2303223 (Exon2)			Somatic Mutation (Exon3)		
	G/G (*n* = 60)	G/A (*n* = 4)	*p*-Value	A/A (*n* = 61)	A/G (*n* = 5)	*p*-Value	No (*n* = 61)	Yes (*n* = 4)	*p*-Value
IRS	5.88 ± 2.73	5.50 ± 4.81	0.8860	5.89 ± 2.80	6.40 ± 4.63	0.8202	5.96 ± 2.85	3.88 ± 1.65	0.0830
submucosal invasion	44.6%	25.0%	0.6261	42.1%	20.0%	0.6398	42.1%	50.0%	1.0000
muscle invasion	21.4%	25.0%	1.0000	21.1%	20.0%	1.0000	19.3%	50.0%	0.1963

The *p*-values were calculated using the Fisher’s Exact test.

**Table 3:** Cases with somatic mutations in exon3 of ZNF668 gene.

	DNA Changes	Protein Changes	Mean_IRS
	pT1≤	pT2≤	
Case A	c.825C>T +	synonymous −	10.5
Case B	c.[910G>T(;)916_931delinsGGAGCTTGGGCATCAA] −	p.[G298T(;)G304_E311delinsWVGAWASK] −	4.0
Case C	c.1339G>C +	p.A447P +	1.5
Case D	c.1556G>T −	p.Q515H −	5.0
Case E	c.[1781A>G(;)1974C>T(;)2028C>G] +	p.H594R +	5.0

pT1≤ and pT2≤ indicate submucosal and muscular invasion, respectively. 1974C>T and 2028C>G in Case103 are located in the 3′-UTR.

## Data Availability

The datasets generated during and/or analyzed during the current study are not publicly available but are available from the corresponding author upon reasonable request.
